# New York Letter

**Published:** 1895-10

**Authors:** Ogden C. Ludlow

**Affiliations:** 2309 South Avenue; New York


### *CORRESPONDENCE.*

### NEW YORK LETTER.

New York, September 10, 1895.

*Atlanta Medical and Surgical Journal:*

Although the treatment of pulmonary tuberculosis by the administration of creosote has been before the medical profession for many years, and has been made almost a routine practice with many physicians, practitioners are not all agreed as to the benefits to be derived from this line of medication. Some lavishly extol the virtues of creosote in consumption, while others assert with equal emphasis that it does little or no good in such cases, and more than this, that it often ruins the digestion of these poor unfortunates, and so deprives them of their mainstay in their grim battle with the bacilli. I think that I but voice a common experience of all, when I say that time and again consumptives, who have "gone the rounds," come to us for aid, but begging not to be given any more creosote. I have even known the relatives of one of these patients to assert, after death had claimed its victory, that "Doctor so-and-so ruined the stomach by pouring into it so much creosote, and so hastened the death."

What answer shall we make to this charge? In a discussion on the value of creosote in pulmonary affections, which took place here at a meeting of the Clinical Society of the Post-Graduate School, there was some diversity of opinion, though the majority distinctly favored the free use of creosote in such cases. Dr. Reynold W. Wilcox said that he had recently had occasion to make a search of the literature, and he had found that in 275 papers published on creosote and its derivatives, there was a striking unanimity of opinion regarding the value of this class of drugs, although the idea that creosote exerted its beneficial action through its germicidal powers had been pretty generally abandoned. The great drawback to its use was, of course, its tendency to cause gastric and renal irritation. The former might be partially avoided by administering the creosote in pills of paraffin, or pills coated with keratin, so that they would not dissolve until they had reached the intestine. He knew of no way of preventing the irritation of the kidneys. Another of the many illustrations of the wide difference between the actions of drugs in the laboratory and at the bedside, is to be found in the fact, that although guaiacol is composed largely of creosote, its therapeutic action is not at all comparable with that of creosote. Dr. Wilcox said that an experience with the carbonate of creosote in the treatment of about three hundred cases of pulmonary tuberculosis, had taught him that this was the best preparation. It should be given in doses of twenty minims, or less, three times a day in a little whisky. He had never known it to excite gastric disturbance, although its administration would sometimes cause a darkening of the urine. In conclusion, he felt that without doubt, creosote had a distinct field of usefulness in the therapeutics of pulmonary tuberculosis. Dr. J. C. Smith said that he had made extensive use of the beechwood creosote, and could now point to six or eight cases of tuberculosis that had been apparently cured by its use. While it had no direct effect on the tubercle bacilli, it promptly diminished the bronchial secretion. He had seen no greater benefit from large than from small doses of the creosote, so that it was his custom to give from one to three minims of the drug with half a drachm each of glycerine and whisky. Dr. Louis Fischer also commented on the favorable influence of the creosote on the accompanying bronchitis, and particularly on the fever and the night-sweats. In one case particularly, the night-sweats had been controlled far better by the creosote than by atropine. He had chiefly used the carbonate of creosote, and greatly preferred it to the other preparations because of its palatability and insolubility in the gastric fluids. Although he had given it to quite young children in as large doses as fifteen minims, three times a day, he had never observed any darkening of the urine. The first discordant note in this general eulogy of creosote was struck by the chairman of the meeting, Dr. William Henry Porter. He said that he had not been able to bring himself to use creosote as extensively as the other speakers had done, because he had been unable to find any chemist who could tell him what creosote really was, and consequently he shrank from introducing any more uncertain factors into the complicated problems of therapeutics that daily confronted the clinician. It should not be forgotten that nine-tenths of the creosote found in the shops was nothing more than crude carbolic acid. Remarkable as were the cases of apparent cure reported by Dr. J. C. Smith, he felt that he could match them with others in which the almost marvelous change had been wrought by nothing more formidable than the judicious use of eggs, milk, and beefsteak. Our object was to improve the general nutrition and vitality of the patient, yet patients have come to him repeatedly after a course of the creosote treatment, with their digestion completely ruined. Those who had advocated the use of creosote carbonate should bear in mind that this preparation is quite insoluble, and there is some doubt about its entering the system at all.

From all of the foregoing I am forced to the conclusion that while there is much benefit to be derived by patients suffering from pulmonary tuberculosis from taking creosote, the administration of this irritating drug is often pushed to the point of interfering with instead of assisting the digestive and nutritive processes, and, as a result, the physician who has mastered the subject of dietetics is able to score better than his professional brother who slavishly adheres to his drugs.

I have given some little space to the consideration of this subject, because it is hard to conceive of a physician, no matter what may be his special field of work, who is not interested in the treatment of consumption. I desire now to touch upon another subject of general interest—cardiac disease as a complication of pregnancy and labor. Dr. Julius Rosenberg brought up this theme for discussion by presenting a paper before the Obstetrical Section of the Academy of Medicine, in which he reported two fatal cases. The first one was that of a primipara, thirty-two years of age, with a loud systolic murmur. As the pains were feeble, and labor tedious, the forceps were applied, and a living child delivered. Shortly after the expression of the placenta, she suddenly became cyanotic, and her pulse irregular; and about three hours afterward she died.

The autopsy showed fatty degeneration of the heart and thickening of the mitral valve. The second patient had previously suffered from flatulent dyspepsia, and was found to have a systolic and a pre-systolic murmur. When about seven months advanced in pregnancy, albuminuria appeared without casts, and her vision was somewhat disturbed. These symptoms were temporarily relieved by placing her on a milk diet and administering small doses of calomel. Labor set in at the beginning of the ninth month, and as her pulse was feeble, and there were considerable edema and cyanosis, the cervix was rapidly dilated, and the child extracted with the forceps. Oxygen gas and cardiac stimulants were given, but there were increasing flatulence and pain over the precordium. Her pulse was about 110 just after the delivery of the placenta. Four hours after confinement there was a sudden convulsive twitching of the face, and she expired. Dr. Rosenberg said that statistics show that a large number of cases in which there are marked mitral or aortic lesion terminate fatally at confinement. When one considers the great variations in the circulation incident to pregnancy and delivery, and also the generally impoverished condition of the blood, it is small wonder that the heart, already seriously crippled by disease, proves unable to meet the strain put upon it. If such cases were seen sufficiently early absolute rest and a milk diet should be enjoined, and if serious symptoms were not yet present the immediate induction of labor might avert a fatal termination. Of course, such treatment should be aided by the administration of the usual cardiac stimulants, and particularly by full doses of the vaso-dilators. In order to avoid any unnecessarily sudden disturbance of the circulation, it would be well not to hasten the delivery of the placenta. In discussing this paper, Dr. Egbert H. Grandin said that in most cases of simple cardiac disease without dilatation, the first and second pregnancies were safely passed through because of adequate cardiac compensation; hence, unless the cardiac lesion were very severe, it seemed to him that marriage was allowable. But just as soon, in a case of this kind, as cardiac dyspnea or albuminuria made its appearance, the physician in attendance should proceed to empty the uterus. This should be done at one sitting, the patient being anesthetized, preferably with chloroform. As the time of greatest anxiety was just after delivery, it behooved the physician to remain at the bedside a longer time than usual. Dr. S. Baruch favored the immediate induction of labor in every case of serious cardiac lesion complicated with pregnancy. Dr. S. Marx said that he had attended four cases of pregnancy complicated with heart disease, and the fact that four of these had recovered he attributed largely to the use of nitroglycerine. This drug, he said, removed congestion, and unlike digitalis and strophanthus, did not act as a stimulant to the kidneys. As ergot was a vaso-constrictor, it should be avoided as far as possible in these cases. In discussing the reason for these cases terminating so suddenly after delivery, and consequently after the heart had successfully stood the strain of pregnancy and of labor, Dr. Mary Putnam-Jacobi said that it was just at this time that thrombosis was well known to be prone to occur. It seemed, therefore, probable that the heart was able to bear up under its burden owing to the extra resistance in the circulation during pregnancy, but that when this resistance was removed by delivery, a condition favorable to the development of stasis in the ventricles of the heart supervened. As so much had been said in the discussion up to this point relative to the extremely bad prognosis of cases in which pregnancy was complicated by organic heart disease, the remarks of Dr. Anna M. Galbraith were of interest. She reported the case of a young primipara, who when first seen was about six months pregnant, and had a double mitral murmur with hypertrophy and dilatation of the heart. She was given saline laxatives with digitalis and the bromides, and under this treatment her dyspnea and cough were greatly improved. There was at no time any albumin in the urine, and she had a normal labor and convalescence. An equally happy result occurred after her second labor, two years later. There were some hemorrhages during both these pregnancies. Dr. Robert A. Murray pointed out that the first danger signals in these cases were edema of the lungs and extremities and cardiac dyspnea, and said that if in spite of this warning the pregnancy were allowed to proceed, it was extremely likely to terminate fatally. The prognosis was still more grave in those cases where there was simply fatty degeneration of the heart without any lesion of the valves. Dr. Rosenberg, in closing this interesting and very practical discussion, said that many cases had been reported in which premature labor had been induced late in pregnancy because of serious cardiac disease, and yet death had speedily closed the scene. He had found nitroglycerine at least of temporary benefit, and while ergot was generally considered to be contraindicated, it might be safely given combined with nitroglycerine. As death from anemia was to be feared, it was not always feasible to dispense with ergot.

OGDEN C. LUDLOW, M.D.

2309 *South Avenue.*

